# Regulatory activity of azabisphosphonate-capped dendrimers on human CD4^+ ^T cell proliferation enhances ex-vivo expansion of NK cells from PBMCs for immunotherapy

**DOI:** 10.1186/1479-5876-7-82

**Published:** 2009-09-24

**Authors:** Damien Portevin, Mary Poupot, Olivier Rolland, Cédric-Olivier Turrin, Jean-Jacques Fournié, Jean-Pierre Majoral, Anne-Marie Caminade, Remy Poupot

**Affiliations:** 1 INSERM, U.563, Centre de Physiopathologie de Toulouse-Purpan, Toulouse, F-31300; Université Paul-Sabatier, Toulouse, F-31400, France; 2 CNRS; LCC (Laboratoire de Chimie de Coordination); 205, route de Narbonne; F-31077 Toulouse, France. Université de Toulouse, UPS, INPT; LCC; F-31077 Toulouse, France

## Abstract

**Background:**

Adoptive cell therapy with allogenic NK cells constitutes a promising approach for the treatment of certain malignancies. Such strategies are currently limited by the requirement of an efficient protocol for NK cell expansion. We have developed a method using synthetic nanosized phosphonate-capped dendrimers allowing such expansion. We are showing here that this is due to a specific inhibitory activity towards CD4^+ ^T cell which could lead to further medical applications of this dendrimer.

**Methods:**

Mononuclear cells from human peripheral blood were used to investigate the immunomodulatory effects of nanosized phosphonate-capped dendrimers on interleukin-2 driven CD4^+^T cell expansion. Proliferation status was investigated using flow cytometry analysis of CFSE dilution and PI incorporation experiments. Magnetic bead cell sorting was used to address activity towards individual or mixed cell sub-populations. We performed equilibrium binding assay to assess the interaction of fluorescent dendrimers with pure CD4^+ ^T cells.

**Results:**

Phosphonate-capped dendrimers are inhibiting the activation, and therefore the proliferation; of CD4^+ ^T cells in IL-2 stimulated PBMCs, without affecting their viability. This allows a rapid enrichment of NK cells and further expansion. We found that dendrimer acts directly on T cells, as their regulatory property is maintained when stimulating purified CD4^+ ^T cells with anti-CD3/CD28 microbeads. Performing equilibrium binding assays using a fluorescent analogue, we show that the phosphonate capped-dendrimers are specifically interacting with purified CD4^+ ^T cells. Ultimately, we found that our protocol prevents the IL-2 related expansion of regulatory T cells that would be deleterious for the activity of infused NK cells.

**Conclusion:**

High yield expansion of NK cells from human PBMCs by phosphonate-capped dendrimers and IL-2 occurs through the specific inhibition of the CD4^+ ^lymphocyte compartment. Given the specificity of the interaction of dendrimers with CD4^+ ^T cell, we hypothesize that regulatory activity may signal through a specific receptor that remains to be indentified. Therefore phosphonate-capped dendrimers constitute not only tools for the *ex-vivo *expansion of NK cells in immunotherapy of cancers but their mode of action could also lead to further medical applications where T cell activation and proliferation need to be dampened.

## Background

Natural Killer cells constitute a heterogeneous and multi-functional population of the innate immune system. Although the CD56^dim/bright ^functional dichotomy has been revised recently [1], NK cells are generally divided in two subsets that differ in their anatomic distribution, cytotoxic potential and ability to proliferate and produce cytokines [2,3]. NK cells initially-obtained their name due to their natural cytotoxicity against tumor cells requiring no prior sensitization, unlike T cells [4]. It is well established that the cytotoxicity of NK cells relies notably on their ability to sense the decrease/absent expression of MHC-I molecules on their target ("missing-self model") [5,6]. In humans, this sensing is controlled by a set of inhibitory receptors belonging to the Killer immunoglobulin-like receptor (KIR) family and/or the heterodimer CD94/NKG2A: each receptor having variable specificity for allotypic variants of MHC-I molecules [7]. The NK cell repertoire of inhibitory receptors is qualitatively and quantitatively variable between humans due to the inherited set of genes coding for these receptors, but also within the same individual, due to the stochastic expression of these genes [8]. This has important implications particularly during the treatment of acute leukemias which require a Stem Cell Transplantation (SCT). Indeed, alloreaction mediated by NK cells could occur between haploidentical individuals presenting a functional mismatch in the NK cell repertoire towards recipients MHC-I ligands. In this context, NK cell alloreactivity has been shown to increase prognosis by enhancing anti-tumor activity (GvL effect) and decrease side effects of immune reconstitution (GvHD) by depleting recipients' DCs [9,10]. In mice, infusion of alloreactive NK cells in the context of SCT also induces potent antitumor effects [9,11] and such therapeutic approaches are now realistic in humans [12]. More generally, adoptive transfer of *ex-vivo *expanded NK cells constitutes a promising approach in immunotherapy of cancer [13,14]. Unfortunately, NK cell expansion remains tedious to achieve, using protocols with purification steps, clonal dilution and/or monoclonal antibodies limiting the outcome of NK cell-based immunotherapy [15]. Dendrimers are versatile tree-like branched synthetic polymers with very promising medical applications such as chemotherapeutic agent delivery [16]. More remarkably, it was shown that a N-acetyl-glucosamine-coated poly-amido-amine (PAMAM) dendrimer stimulates an antitumor immune response involving enhancement of the functions of CD4 T cells and NK cells [17]. A mannosylated dendrimer of the same PAMAM family conjugated to ovalbumin (OVA) has been shown to induce, *in vitro *and *in vivo*, a very potent immune response against OVA highlighting their adjuvanticity [18]. We have recently reported that a group of nanosized synthetic phosphonate-capped dendrimers (especially **3a-G1**) activate human monocytes toward an anti-inflammatory and immunosuppressive pathway [19-21]. We also described an innovative protocol using dendrimer **3a-G1 **that allows high yield expansion human NK cells from PBMCs [22]. Expanded NK cells are fully functional and can efficiently lyse a broad spectrum of tumor cell lines. Prospecting the transfer from bench to clinic of such expanded NK cells, we had to decipher the origin of this expansion process. Here, we show that **3a-G1 **driven expansion of NK cells from PBMCs is not occurring through a direct activation of the NK cell reservoir but actually through the regulation of CD4^+ ^T cell expansion. Ultimately, we found that our protocol prevents the IL-2 related expansion of CD4^+^/CD25^+^/CD127^low^/FoxP3^+ ^regulatory T cells. Given the fact that regulatory T cells might affect NK cell functions *in vivo *[23,24], this last finding supports the use of our expansion protocol for NK cell-based adoptive immunotherapy of cancers.

## Methods

### Blood samples, cells and cell cultures

Fresh blood samples were collected from healthy adult donors, and PBMCs were prepared on a Ficoll-Paque density gradient (Amersham Biosciences AB, Uppsala, Sweden) by centrifugation (800 g, 30 min at room temperature). Collected PBMCs were washed twice and finally diluted at 1.5 million cells/ml in complete RPMI 1640 medium, i.e., supplemented with penicillin and streptomycin, both at 100 U/ml (Cambrex Bio Science, Verviers, Belgium), 1 mM sodium pyruvate, and 10% heat-inactivated fetal calf serum (both from Invitrogen Corporation, Paisley, UK) and when specified recombinant IL-2 (400 U/ml) and dendrimers solution (20 μM). Detailed chemical synthesis of dendrimers could be found here [19,20,22]. NK cells, CD4 T cells, and monocytes were selected from PBMC by magnetic cell sorting using respectively the NK isolation kit II, the CD4 T cell isolation kit and CD14 microbeads (Miltenyi Biotec, Auburn, CA, USA) according to manufacturer's recommendations. Cell purity checked by flow cytometry was always >95% for NK cells and >98% for CD4 T cells and monocytes.

### Flow cytometry and cell surface staining

Flow cytometry was performed using a LSR-II cytometer, BD biosciences, San Jose, CA, USA. Data treatment and analysis were performed using Flowjo or BD FacsDiva software. Anti-CD3 FITC or PE (UCHT1), anti-CD4 PE or PC5 (13B8.2), anti-CD56 PC5 (N901), anti-CD127 PE (R34.34) (Beckman Coulter Immunotech), anti-CD14 PE or PC7 (clone M5E2), anti-CD56 PC7 (clone B159) (BD biosciences) and anti-FoxP3 PE (PCH101) (eBioscience) were used according to manufacturer's recommendations. For surface staining, cells were incubated with fluorochrome-conjugated monoclonal antibodies in cold PBS containing 5% of fetal bovine serum at 4°C for 15 min in the dark, then washed before analysis. Eventually, intracellular staining of FoxP3 was done using Foxp3 Staining Buffer Set (eBioscience) following manufacturer's instructions.

### CFSE dilution, NK cell amplification and cell cycle analysis

For carboxyfluorescein succinimidyl ester (CFSE) cell staining, a 250 μM stock solution in DMSO was freshly diluted in PBS and immediately used to resuspend cells at 5.10^6 ^cells/ml for 8 min at 37°C. Reaction was stopped after adding one volume of fetal calf serum and cells were washed twice with PBS before culture. For anti-CD3/CD28 stimulation of PBMCs or purified CD4^+ ^T cells, 5.10^4 ^CFSE labelled cells were mixed 1.2.10^3 ^anti-CD3/CD28 mAb-coated Dynabeads (Invitrogen) and displayed in U-shaped 96 well plates. CFSE dilution was favourably analyzed after 7 days of culture. In experiments aimed at measuring the NK cell amplification, cultures were maintained during 12 to 14 days to enhance the effect of the inhibition of CD4^+ ^T cell proliferation on the subsequent amplification of NK cells.

For cell cycle analysis, 10^5 ^cells were resuspended on ice with cold PBS containing 2% fetal calf serum and fixed with 3 volumes of absolute ethanol overnight at 4°C. Pelleted cells were resuspended with 50 μl propidium iodide 10 μg/ml in PBS and 18 μl of a RNAse solution for 30 min RT and washed with PBS containing 5% fetal calf serum before flow cytometry analysis.

### Equilibrium binding assay

Cells in triplicates were incubated for 15 min on ice with detailed concentration of dendrimer solution in PBS containing 5% fetal calf serum and washed before flow cytometry analysis. Progression of cellular mean fluorescence intensity was analysed using modelling software (SAAMII, v1.2, University of Washington).

### Statistical analysis

Statistical analyses were carried out using the biostatistic software GraphPad Prism (GraphPad Software, Inc). Wilcoxon signed-rank test was performed to compare amplification rate and cell proportion between 3a-G1 treated and untreated samples (*: P ≤ 0.05, **: P ≤ 0.01, ***: P ≤ 0.001).

## Results

### Azabisphosphonate branched dendrimers specifically inhibit IL-2 driven proliferation of CD4^+ ^T cell among human PBMCs

**Figure 1:**
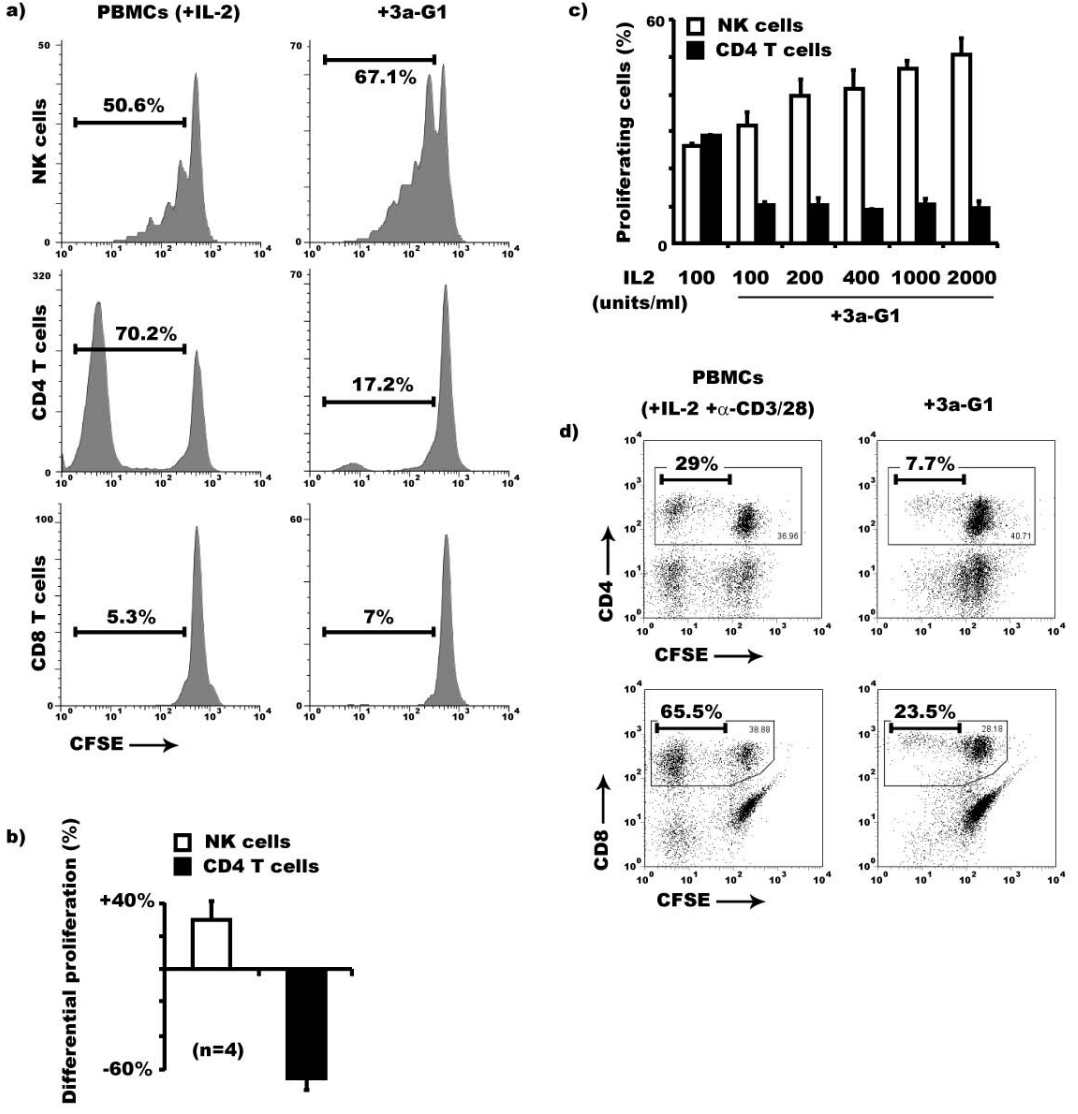
**Dendrimer 3a-G1 selectively inhibits CD4^+ ^T cell proliferation among IL-2 cultured PBMCs during the first week of culture**. a) Among PBMCs, NK and CD4^+ ^T cells are the two major cell populations which spontaneously proliferate in response to IL-2 during the first week of culture. **3a-G1 **not only enhances the proliferation of NK cells but it also affects the capacity of the CD4^+ ^T cell population to proliferate. b) Average NK cell proliferation increased 29.4% ± 12.1% while CD4^+ ^T cell proliferation decreased 66.1% ± 7.03% in **3a-G1 **treated cultures compared to those containing only IL-2 (Day 7, n = 4). c) Impaired proliferation of CD4^+ ^T cells in the presence of **3a-G1 **is not rescued by higher IL-2 concentration after a week of culture. Results representative of two independent experiments performed on two individual donors. d) CD8^+ ^T cell proliferation was induced adding anti-CD3/CD28 coated beads on IL-2 cultured PBMCs. The percentages indicated are expressed after gating on the relevant CD4^+ ^or CD8^+ ^T cell population. Addition of **3a-G1 **in these conditions affected CD4^+ ^as well as CD8^+ ^T cell proliferation.

We have previously reported that addition of azabisphosphonate capped dendrimers (**3a-G1**) on human PBMCs together with human recombinant IL-2 allows a massive *ex-vivo *expansion of fully functional CD3^-^/CD56^+ ^NK cells within four weeks of culture [22]. In order to elucidate the short term events leading to this selective expansion process, we intuitively hypothesised a direct stimulation of NK cells by dendrimers which would induce their selective proliferation. Then, using freshly isolated human PBMCs, we performed a CFSE dilution experiment to address cell division of the different cell populations after 7 days. Unsurprisingly, when gating on the CD3^-^/CD56^+ ^NK cell population, we observed a reproducible slight increase in the proportion of divided NK cells in the presence of **3a-G1 **(Fig. 1a). But a more striking effect was unexpectedly observed when gating on CD3^+^/CD4^+ ^T cells. Indeed, expansion of some CD4^+ ^T cells is always observed when PBMCs are cultured with IL-2 alone. In contrast, this is not happening when **3a-G1 **is present. We assessed the reproducibility of this phenomenon by performing the same experiment over four independent healthy donors. Results showed an average inhibition of CD4^+ ^T cell proliferation of 66 ± 7% versus a mean increase of 29 ± 12% of NK cell proliferation, when cultured with **3a-G1 **and IL-2 in comparison with IL-2 alone (Fig. 1b). Being consumed by both cell types, we rejected the possibility of a competition for IL-2 by performing the same assay at various concentrations of the cytokine. Irrespective of IL-2 concentration, **3a-G1 **locks CD4^+ ^T cell proliferation. In contrast, NK cell proliferation increased gradually from 31.2% to 50.4% as it did in the absence of dendrimers (Fig. 1c and data not shown). In parallel, we also followed CD8^+ ^T cell, γδ T cell, NK T cell and B cell counts observing that these cells are persisting similarly in both culture conditions excluding the possibility of apoptosis induction of these populations by dendrimers, excepting B cells that died within the first days of culture even in the absence of dendrimers (Data not shown). Given the fact that **3a-G1 **inhibits CD4^+ ^T cell proliferation without affecting NK cell one within PBMCs, we checked whether this activity could not be broadened to all T cells. When stimulating T cell proliferation adding anti-CD3/CD28 coated beads to CFSE labelled PBMCs, we induced CD4^+ ^and CD8^+ ^T cell proliferation (Fig. 1d). Interestingly, when adding **3a-G1**, CFSE diluted events were strongly reduced within both T cell subsets indicating that although CD8^+ ^T cells are not a major proliferative population in IL-2 cultured PBMCs, dendrimer **3a-G1 **may also inhibits their expansion in other conditions.

### *3a-G1 *interferes with CD4^+ ^T cell activation and proliferation inducing NK cell enrichment

**Figure 2:**
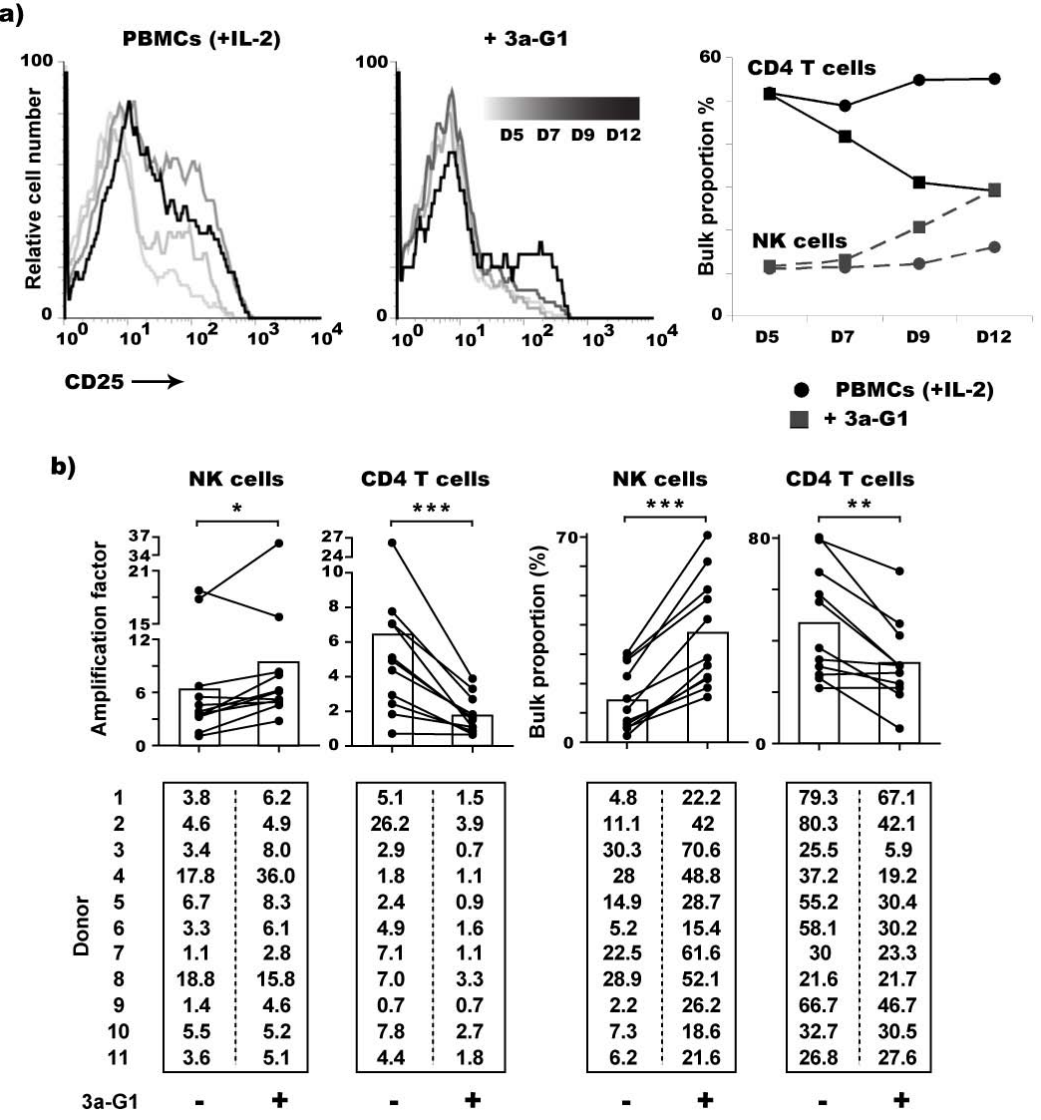
**3a-G1 treated PBMCs show progressive enrichment in NK cells at CD4^+ ^T cell expense during the second week of culture**. a) CD25 expression gated on CD4^+ ^T cells (left graphs) and NK cell versus CD4^+ ^T cell proportion at days 5, 7, 9 and 12 of culture (right graph). b) Amplification factor (left) and proportion (right) of NK and CD4^+ ^T cell populations among PBMCs from eleven different donors after 12 to 14 days treatment with **3a-G1**. Histograms indicate the means of the data collected from the eleven donors (Wilcoxon signed rank t test, *: P ≤ 0.05, **: P ≤ 0.01, ***: P ≤ 0.001).

Focusing our analysis on CD4^+ ^T cells, we looked for the surface expression of the α-chain of the IL-2 receptor, CD25, a transient marker of T cell activation after 5, 7, 9 and 12 days of culture (Fig. 2a). Correlating with their proliferation status described above, CD25 surface expression is rapidly acquired by some CD4^+ ^T cells when PBMCs are cultured with IL-2 alone, however this is markedly delayed when **3a-G1 **is present. Interestingly, the percentage of CD4^+ ^T cells and NK cells during this period of culture remains constant when PBMCs are cultured with IL-2 alone. In contrast and reproducibly over 11 independent donors, the NK cell proportion progressively increases in the presence of **3a-G1 **while the CD4^+ ^T cell proportion decreases during the first two week of culture (Fig. 2b). Natural CD4^+ ^T cell predominance among PBMCs is decreased significantly when cultured with **3a-G1 **(46.7 ± 22% versus 31.3 ± 16.1%) giving a very significant advantage to NK cells (14.7 ± 10.8% versus 37.1 ± 18.9%). Remarkably, amplification factor means of each subset are very close when PBMCs are cultured with IL-2 alone (6.36 ± 6.11 for NK cells versus 6.4 ± 6.96 for CD4^+ ^T cells). However, for ten of eleven donors, NK cell expansion was significantly enhanced by the presence of **3a-G1**. Conversely, the addition of **3a-G1 **to cultures induces a massive and significant reduction of the expansion of CD4^+ ^T cells. At the donor level, a higher proportion of NK cells tend to be associated, in absence or in presence of **3a-G1**, with a low proportion of CD4 T cells within the same donor and vice versa. This clearly reflects a competition between NK and CD4 T cell on which **3a-G1 **seems to be acting. Therefore, halfway through the expansion procedure, **3a-G1 **inhibits T cell activation, their maintenance, and consequently favours the representation and then further expansion of NK cells driven by IL-2.

### Regulatory activity of *3a-G1 *is direct and does not require monocytes

**Figure 3:**
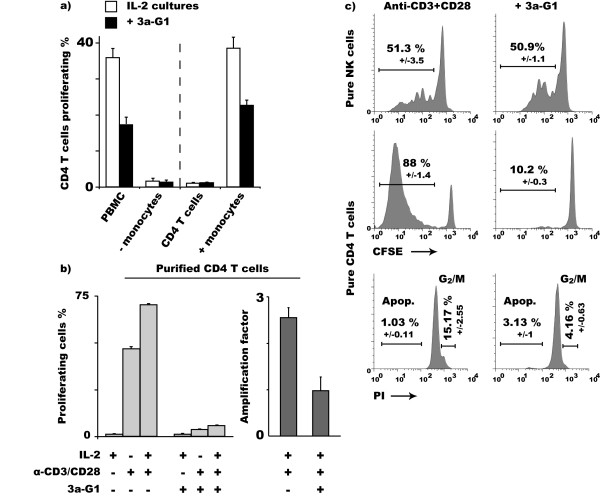
**Regulatory activity of 3a-G1 upon CD4^+ ^T cell proliferation is direct and T cell restricted**. a) CFSE dilution of CD4^+ ^T cells within IL-2 treated CFSE labelled PBMCs or depleted of monocytes (Right), CFSE dilution of CFSE labelled purified CD4^+ ^T cells ± **3a-G1 **± autologous monocytes (Ratio 5:1). b) Regulatory activity of dendrimers is not mediated by autologous monocytes as **3a-G1 **also inhibits CFSE dilution of purified CD4^+ ^T cells stimulated with anti-CD3/CD28 coated beads. c) Regulatory activity of **3a-G1 **is restricted to T cells as under the same conditions IL-2 stimulated proliferation of autologous NK cells is not affected. Cell cycle analysis shows that the decrease of proliferation of **3a-G1 **treated CD4^+ ^T cells correlates with a reduction of mitotic events.

We previously reported that phosphorus-containing dendrimers are rapidly taken up by monocytes leading to their activation [19,20]. To evaluate the link between this effect and the impaired proliferation/expansion of CD4^+ ^T cells, we extended our CFSE dilution assay using monocyte-depleted PBMCs. In the absence of monocytes, the proliferation of purified CD4^+ ^T cells is abrogated; therefore monocytes are required for the priming of autologous T cell proliferation. Co-culturing monocytes with previously purified and CFSE labelled autologous CD4^+ ^T cells (1:5 ratio), the priming of the T cell proliferation was recovered and the inhibition by **3a-G1 **of the subsequent proliferation maintained (Fig. 3a). In parallel, we also stimulated CFSE labelled CD4^+ ^T cells with anti-CD3/CD28 coated beads. In such conditions, the capacity of **3a-G1 **to regulate the proliferation and the expansion of T cells was maintained in the presence or absence of exogenous IL-2 (Fig 3b). Thus, monocytes are involved in the *ex-vivo *priming of autologous CD4^+^T cells but **3a-G1 **is directly acting on CD4^+^T cells to regulate their proliferation. **3a-G1 **regulatory activity was also observed using 50 ng/ml PHA as a stimulus for the proliferation of pure CD4^+^T cells (data not shown). In contrast, proliferation of purified autologous NK cells was neither enhanced nor impaired when grown under the same conditions, i.e. IL-2 + anti-CD3/CD28 coated beads, +/- **3a-G1 **(Fig. 3c). In order to reject the possibility that our CFSE analysis could be biased by the exclusion of dead cells from the morphological gate, we checked that **3a-G1 **does not induce apoptosis of CD4^+ ^T cell. We performed propidium iodide nuclear staining on purified CD4^+ ^T cells stimulated for 7 days with anti-CD3/CD28 micro-beads and looked at the proportion of cells in the G1 or G2/M phase of mitosis versus cells undergoing nucleus fragmentation. A very slight increase in the percentage of apoptotic cells was observed when cells were cultured with **3a-G1 **but most of the cells maintained their DNA integrity. Conversely, the proportion of mitotic events were reduced by 72% (15.8% to 4.2%) (Fig. 3c, bottom). Given the fact that **3a-G1 **by itself is able to inhibit the proliferation/expansion of CD4^+ ^T cells, while not affecting the viability of these cells, highlights an unsuspected regulatory property of **3a-G1 **molecules on human CD4^+ ^T cells.

### Cellular interaction of azabisphosphonate branched dendrimers using a fluorescent analogue of *3a-G1*

**Figure 4:**
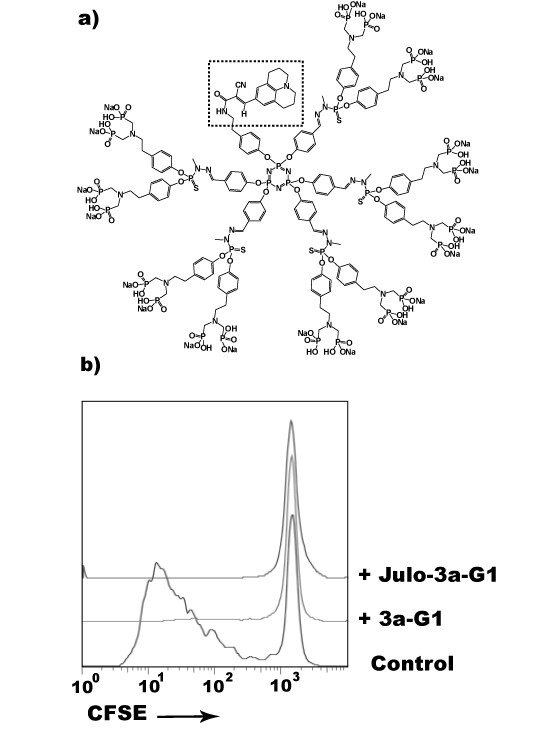
**Julolidine analogue of 3a-G1 presents constant regulatory activity on CD4^+ ^T cell proliferation**. a) Detailed structure of the julolidine analogue of **3a-G1**. Dashed frame highlights the julolidine moiety that has replaced one of the azabisphosphonate claws of the parental **3a-G1 **dendrimer. b) The replacement of one azabisphosphonate branch by the julolidine unit does not alter the capacity of the fluorescent **3a-G1 **analogue to inhibit CD4^+ ^T cell proliferation under anti-CD3/CD28 stimulation.

**Figure 5:**
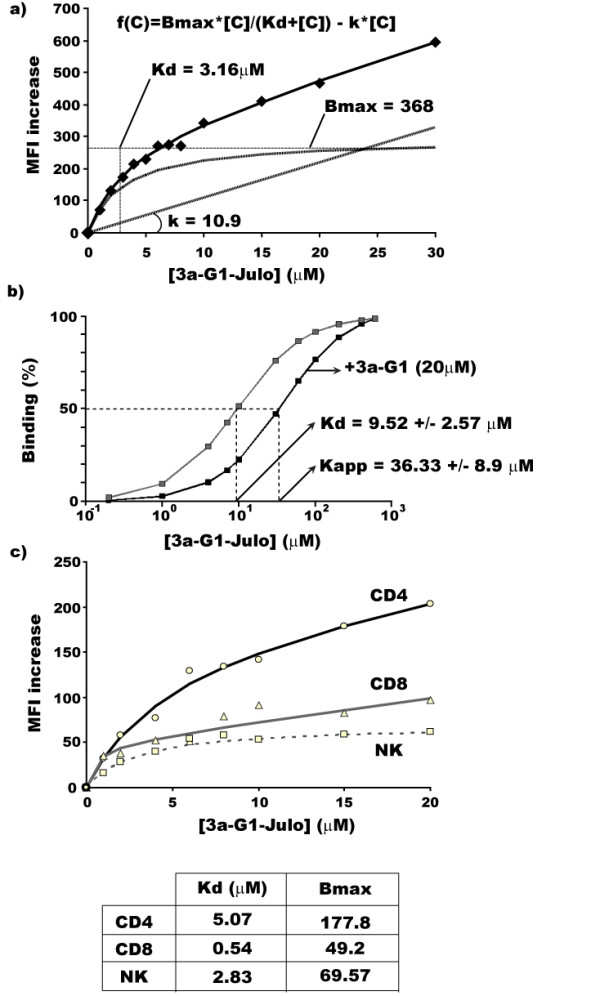
**Specific and competitive interaction of azabisphonate dendrimers with pure CD4^+ ^T cells**. a) Equilibrium binding curve (dots) and equation of the two-component binding interaction after software modelling (Values of the constants are detailed on the graph). b) Competition with 20 μM **3a-G1 **increases Kd showing that both dendrimers are competing for same binding sites. c) Equilibrium binding curve of Julo-**3a-G1**, Kd and Bmax, comparing CD4, CD8 T cells and NK cells using monocyte depleted PBMCs.

To further analyze the cellular interaction of phosphonate-capped dendrimers, we used an analogue of the **3a-G1 **in which one of the branches of the dendrimer was replaced during synthesis with a fluorescent moiety, the julolidine, leading to the **3a-G1**-Julo [20]. Addition of the fluorescent derivatives on purified CD4^+ ^T cells stimulated by anti-CD3/CD28 micro-beads revealed that proliferation was still strongly inhibited 3.6% ± 0.2% compared to 67.5% ± 5.9% in the control conditions (Fig. 4). Performing an equilibrium binding assay coupled with flow cytometry analysis, we revealed a specific interaction signature of **3a-G1**-Julo with purified CD4^+ ^T-cells. After incubation with increasing concentration of **3a-G1**-Julo, we observed an increase in the mean fluorescence intensity of the cells, indicating a progressive labelling of the cells (Fig. 5a). However, the fluorescence signal never reached a clear saturation step. Moreover, at low concentration, the staining curve increased faster than at higher concentration, indicating a two-component binding interaction. Indeed, using a root mean square minimization analysis and the Akaike criterion cut-off, we found that the best model resulted from the addition of a specific and saturable fixation component in one hand and a linear and non-specific component fixation in the other hand, according to the equation: f(C) = Bmax*C/(Kd+C) + k*C where Bmax reflects the relative cell binding capacity, C the concentration of the **3a-G1**-Julo, Kd is the dissociation constant and k the coefficient of the non-specific fixation component. Interestingly, competition experiments revealed that the parental **3a-G1 **was able to shift the apparent dissociation constant (Kapp) of **3a-G1**-Julo without affecting Bmax (Fig. 5b), and vice versa (data not shown), meaning that both dendrimers are competing for the same binding sites. Therefore, CD4^+ ^T cells are expressing receptors that specifically interact with phosphonate-capped dendrimers. Interestingly, we noticed that these receptors are linked to some extent to T cell proliferation as anti-CD3/CD28 activated T cells have a significantly lower Kd than resting autologous T cells (Fig. 5a and 5b). Knowing that dendrimers are not only interacting with CD4 T cells but also monocytes [19,20] and given the fact that **3a-G1 **is also able to inhibit CD8 T cell proliferation (Fig. 1), we performed the same equilibrium binding experiments on monocyte depleted PBMCs to study whether **3a-G1 **could interact with other lymphocytes sub-populations. As shown in Fig. 5c, we can also detect a specific interaction of Julo-**3a-G1 **with CD8 T cells and NK cells. We found some differences in the Bmax reflecting different level of expression of receptor(s) for **3a-G1 **ligands but more interestingly some variation in the dissociation constant value which would indicate that these receptors may be different for each sub-population.

### *3a-G1 *inhibits IL-2 related expansion of CD4^+^/CD25^+^/CD127^-^/FoxP3^+ ^regulatory T cells

**Figure 6:**
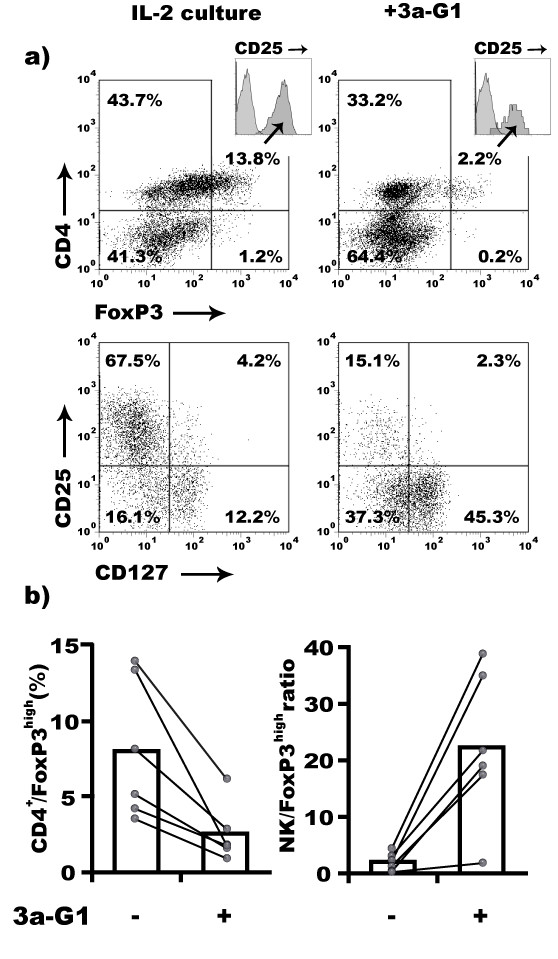
**3a-G1 prevents IL-2 driven expansion of CD4^+^/Foxp3^high ^regulatory T cells**. a) Expanded CD4^+^/CD25^+ ^T cells among IL-2 treated PBMCs present characteristics of regulatory T cells, e.g. CD127^-/low ^and FoxP3^high^. **3a-G1 **interferes with the expansion of these cells. Markers analysed in upper quadrants are obtained after gating on lived cells based on Forward/Side scatter signal. Insert of CD25 staining is gated on FoxP3^+ ^cells overlaid with isotypic control antibody staining. CD25/CD127 quadrant is gated on CD4^+ ^T cells. Percentage of cells from parental gate is indicated in each quadrant. b) Increased ratio of NK:FoxP3^high ^T cells during **3a-G1 **driven expansion of human NK cells from PBMCs.

IL-2 is critical for the *ex-vivo *expansion of suppressive regulatory cells [25]. Using high doses of IL-2 in our NK cell expansion protocol, we were interested in whether regulatory T cells could persist and even expand in these conditions, thus dampening the overall efficacy of **3a-G1 **expanded NK cells [23]. We found indeed that the IL-2 level in the control conditions favours the activation of T cells that are FoxP3^+ ^and that express high levels of CD25 and low level of CD127, the phenotype of regulatory T cells [26]. In contrast, **3a-G1**-treated PBMCs contain a markedly reduced proportion of these cells (Fig. 6a). We accumulated such evidence over six different donors and then estimated the proportion of CD4^+^/FoxP3^high ^cells vs. NK cells in both conditions. For all donors **3a-G1 **prevented the generation of regulatory T cells and together with higher NK cell proportion, it dramatically increased the ratio between these two subsets (Fig. 6b).

## Discussion

In this report, we elucidate the origin of the enrichment and subsequent expansion of NK cells from human PBMCs using **3a-G1 **phosphonate-capped dendrimers [22]. Therefore, we focused our analysis on the first two weeks of culture although the expansion procedure requires 4 weeks to provide suitable amounts of cells for clinical purposes. Such amplified NK cells are perfectly cytotoxic against the K562 cell line but also a broad range of other tumor cell line. Although this has not been checked systematically, we did found that mid-term amplified NK are also cytotoxic against the K562 cell line and that **3a-G1 **doesn't affect their cytotoxicity when compared with untreated cells [see Additional file 1]. Contrary to expectation, we could not demonstrate any significant activation of proliferation of pure NK cells exposed to **3a-G1**. Conversely, we showed that during the first week of culture, **3a-G1 **mainly acts by inhibiting CD4^+ ^T cell proliferation without affecting NK cells. In terms of cell expansion, we found that NK cells are normally competing with CD4^+ ^T cells when PBMCs are exposed to interleukin-2 and that **3a-G1 **cancels this competition. Therefore, the decreased CD4^+ ^T cell representation results in more nutrients and cytokines for the expansion of NK cells. We propose that the higher proliferation status of NK cells when PBMCs are exposed to **3a-G1 **(Fig. 1) is mainly due to an increase in the availability of IL-2 that has not been consumed by proliferating T cells. Supporting our hypothesis, other investigators have described the use of anti-CD3 antibodies and IL-2 as a method for the *in vitro *expansion of human NK cells from PBMCs [27]. No clues were provided about the origin of this process but it suggests that targeting T cells to some extent sustains the expansion of NK cells from PBMCs. Interestingly; we demonstrated that like such antibodies, **3a-G1 **dendrimers specifically interacts with CD4^+ ^T cells. We believe that this interaction might drive the inhibition of CD4^+ ^T cell proliferation observed not only among PBMCs but also when pure CD4^+ ^T cells were stimulated with anti-CD3/CD28 coated beads. Molecular determinants are still needed regarding the mode of action of **3a-G1 **but given its structural features, it is tempting to speculate that **3a-G1 **could act by triggering Sphingosine 1-phosphate (S1P) receptors. Indeed, there is some evidence that S1P regulates T cell proliferation [28]. Interestingly, the phosphate moiety was shown to be important for this effect. To address that point, we are now concentrating our effort in the synthesis of a biotin analogue of **3a-G1 **to perform pull-down experiment on CD4^+ ^T cell protein extracts with the aim of identifying by proteomics the molecular determinants of **3a-G1 **regulatory activity. Furthermore, Miller and colleagues have described the importance of monocytes in the expansion of human NK cells from IL-2 treated PBMCs [29]. We have shown that depleting monocytes from PBMCs prevents CD4^+ ^T cell proliferation. In agreement with Miller's report, we also found that NK cells are less able to proliferate when monocytes are depleted from PBMCs. Therefore, monocytes are supporting the *ex-vivo *expansion of both cell types. Interestingly, we showed that monocytes rapidly engulfed phosphorus-containing dendrimers and consequently become activated [19,20]. We have addressed the particular mode of activation of these monocytes highlighting an immune-suppressive phenotype on mixed leukocyte reaction [21] that could sustain the inhibition of T cell proliferation although we have shown here, using anti-CD3/CD28 microbeads, that monocytes are not required for regulatory activity of phosphonate-capped dendrimers. Again, Miller and colleagues showed that CD5^+ ^and CD8^+ ^cell depletion led to higher NK cell expansion yield providing support that T cells constitute a barrier for the expansion of NK cells. IL-2 stimulation of PBMCs was shown to elicit absolute expansion of NK cells and CD56^+ ^T cells, e.g. NK-T cells, γδ T cells and some αβ/CD8^+ ^T cells [30]. The combination of IL-2 and **3a-G1 **in our hands also led to a generally slightly higher representation of γδ-T cells (data not shown) but we were never able to detect any NKT (Vα 24^+^) cell or CD8^+ ^T cell expansion under our conditions. In contrast, we found that a proportion of CD4^+ ^T cells that became activated under IL-2 stimulation were presenting a regulatory T cell phenotype e.g. CD25^+^/FoxP3^+^/CD127^*low*^, the best up to date combination to characterise regulatory T cells [26]. Such in vitro induction of T regulatory activity by stimulated human CD4^+^/CD25^- ^has already been described [31]. *In vivo*, regulatory T cells play an important role in maintaining peripheral tolerance and preventing auto-immunity but they could also affect anti-tumor immunity by notably acting on NK cell activity [23,24]. Then, the presence of regulatory T cells during the process of NK cell expansion by **3a-G1 **would have had a highly detrimental effect. Interestingly, the inhibition of CD4^+ ^T cell activation by **3a-G1 **is global and also affects the accumulation of these phenotypically related regulatory T cells. Although it can't be excluded that the presence of few remaining regulatory T cells could have a detrimental effect for the activity of infused NK cells *in vivo*, it does not affect the cytotoxic property of the expanded NK cells *in vitro *against classical tumor cell lines [22].

On the wave of tetramer technology [32], this project was initiated to use dendrimer plasticity to chemically build a platform of bi-phosphate entities that would promote γδ-T cell expansion [33]. This contemporary attempt of building and testing a sophisticated hypothesis actually ended with unexpected results. Indeed, it turned to favour the expansion of NK cells another subset of cytotoxic lymphocytes and we show here that this is happening by selectively inhibiting the activation and proliferation of CD4^+ ^T lymphocytes. Having set the expansion protocol using good manufacturing practice (GMP)-compliant components, we are now planning to translate from bench to clinic the use of such *ex-vivo *amplified NK cells as a conditioning treatment for patients undergoing leukemia therapy. The therapeutic relevance of our method does have some limitation as we did observe variation in NK cell expansion between donors at the term of the amplification process [22]. However, deciphering the molecular determinants of phosphonate-capped dendrimer activity in the regulation of T cell proliferation could also lead to further applications in the treatment of pathologies where T cell proliferation is undesirable, such as cutaneous T-cell lymphoma and/or auto-immune diseases for which efficient treatments are still needed [34].

## Abbreviations used

PBMCs: Peripheral Blood Mononuclear Cells; CFSE: CarboxyFluorescein Succinimidyl Ester; PI: Propidium Iodide.

## Competing interests

The authors declare that they have no competing interests.

## Authors' contributions

DP carried out biological studies and experiments and wrote the manuscript. MP performed biological experiments. OR synthesized the dendrimers used in this study. COT designed and synthesized the dendrimers. JJF designed biological experiment. JPM supervised chemical achievements. AMC designed dendrimers and supervised chemical achievements. RP designed and supervised biological studies, coordinated the study and wrote the paper. All authors have read and approved the final manuscript.

## Supplementary Material

Additional file 1 **3a-G1 does not affect NK cell cytotoxicity**. Standard 4 h ^51^Cr-release assay determining the specific lysis of K562 pulsed cells by PBMCs cultured for two weeks in the presence or in the absence of 3a-G1. Effector/Target ratio was normalized according to the percentage of NK cell present in each culture. Click here for file

## References

[B1] Anfossi N, Andre P, Guia S, Falk CS, Roetynck S, Stewart CA, Breso V, Frassati C, Reviron D, Middleton D (2006). Human NK cell education by inhibitory receptors for MHC class I. Immunity.

[B2] Cooper MA, Fehniger TA, Caligiuri MA (2001). The biology of human natural killer-cell subsets. Trends Immunol.

[B3] Poli A, Michel T, Theresine M, Andres E, Hentges F, Zimmer J (2009). CD56bright natural killer (NK) cells: an important NK cell subset. Immunology.

[B4] Kiessling R, Klein E, Wigzell H (1975). "Natural" killer cells in the mouse. I. Cytotoxic cells with specificity for mouse Moloney leukemia cells. Specificity and distribution according to genotype. Eur J Immunol.

[B5] Karre K (2002). NK cells, MHC class I molecules and the missing self. Scand J Immunol.

[B6] Karre K (2008). Natural killer cell recognition of missing self. Nat Immunol.

[B7] Lanier LL (2005). NK cell recognition. Annu Rev Immunol.

[B8] Uhrberg M (2005). Shaping the human NK cell repertoire: an epigenetic glance at KIR gene regulation. Mol Immunol.

[B9] Ruggeri L, Capanni M, Urbani E, Perruccio K, Shlomchik WD, Tosti A, Posati S, Rogaia D, Frassoni F, Aversa F (2002). Effectiveness of donor natural killer cell alloreactivity in mismatched hematopoietic transplants. Science.

[B10] Ruggeri L, Mancusi A, Capanni M, Urbani E, Carotti A, Aloisi T, Stern M, Pende D, Perruccio K, Burchielli E (2007). Donor natural killer cell allorecognition of missing self in haploidentical hematopoietic transplantation for acute myeloid leukemia: challenging its predictive value. Blood.

[B11] Lundqvist A, McCoy JP, Samsel L, Childs R (2007). Reduction of GVHD and enhanced antitumor effects after adoptive infusion of alloreactive Ly49-mismatched NK cells from MHC-matched donors. Blood.

[B12] Miller JS, Soignier Y, Panoskaltsis-Mortari A, McNearney SA, Yun GH, Fautsch SK, McKenna D, Le C, Defor TE, Burns LJ (2005). Successful adoptive transfer and in vivo expansion of human haploidentical NK cells in patients with cancer. Blood.

[B13] Malmberg KJ, Bryceson YT, Carlsten M, Andersson S, Bjorklund A, Bjorkstrom NK, Baumann BC, Fauriat C, Alici E, Dilber MS, Ljunggren HG (2008). NK cell-mediated targeting of human cancer and possibilities for new means of immunotherapy. Cancer Immunol Immunother.

[B14] Ruggeri L, Mancusi A, Perruccio K, Burchielli E, Martelli MF, Velardi A (2005). Natural killer cell alloreactivity for leukemia therapy. J Immunother.

[B15] Alici E, Sutlu T, Bjorkstrand B, Gilljam M, Stellan B, Nahi H, Quezada HC, Gahrton G, Ljunggren HG, Dilber MS (2008). Autologous antitumor activity by NK cells expanded from myeloma patients using GMP-compliant components. Blood.

[B16] Medina SH, El-Sayed ME (2009). Dendrimers as Carriers for Delivery of Chemotherapeutic Agents. Chem Rev.

[B17] Vannucci L, Fiserova A, Sadalapure K, Lindhorst TK, Kuldova M, Rossmann P, Horvath O, Kren V, Krist P, Bezouska K (2003). Effects of N-acetyl-glucosamine-coated glycodendrimers as biological modulators in the B16F10 melanoma model in vivo. Int J Oncol.

[B18] Sheng KC, Kalkanidis M, Pouniotis DS, Esparon S, Tang CK, Apostolopoulos V, Pietersz GA (2008). Delivery of antigen using a novel mannosylated dendrimer potentiates immunogenicity in vitro and in vivo. Eur J Immunol.

[B19] Poupot M, Griffe L, Marchand P, Maraval A, Rolland O, Martinet L, L'Faqihi-Olive FE, Turrin CO, Caminade AM, Fournie JJ (2006). Design of phosphorylated dendritic architectures to promote human monocyte activation. Faseb J.

[B20] Rolland O, Griffe L, Poupot M, Maraval A, Ouali A, Coppel Y, Fournie JJ, Bacquet G, Turrin CO, Caminade AM (2008). Tailored control and optimisation of the number of phosphonic acid termini on phosphorus-containing dendrimers for the ex-vivo activation of human monocytes. Chemistry.

[B21] Fruchon S, Poupot M, Martinet L, Turrin CO, Majoral JP, Fournie JJ, Caminade AM, Poupot R (2009). Anti-inflammatory and immunosuppressive activation of human monocytes by a bioactive dendrimer. J Leukoc Biol.

[B22] Griffe L, Poupot M, Marchand P, Maraval A, Turrin CO, Rolland O, Metivier P, Bacquet G, Fournie JJ, Caminade AM (2007). Multiplication of human natural killer cells by nanosized phosphonate-capped dendrimers. Angew Chem Int Ed Engl.

[B23] Ghiringhelli F, Menard C, Terme M, Flament C, Taieb J, Chaput N, Puig PE, Novault S, Escudier B, Vivier E (2005). CD4+CD25+ regulatory T cells inhibit natural killer cell functions in a transforming growth factor-beta-dependent manner. J Exp Med.

[B24] Ralainirina N, Poli A, Michel T, Poos L, Andres E, Hentges F, Zimmer J (2007). Control of NK cell functions by CD4+CD25+ regulatory T cells. J Leukoc Biol.

[B25] Thornton AM, Donovan EE, Piccirillo CA, Shevach EM (2004). Cutting edge: IL-2 is critically required for the in vitro activation of CD4+CD25+ T cell suppressor function. J Immunol.

[B26] Liu W, Putnam AL, Xu-Yu Z, Szot GL, Lee MR, Zhu S, Gottlieb PA, Kapranov P, Gingeras TR, Fazekas de St Groth B (2006). CD127 expression inversely correlates with FoxP3 and suppressive function of human CD4+ T reg cells. J Exp Med.

[B27] Carlens S, Gilljam M, Chambers BJ, Aschan J, Guven H, Ljunggren HG, Christensson B, Dilber MS (2001). A new method for in vitro expansion of cytotoxic human CD3-CD56+ natural killer cells. Hum Immunol.

[B28] Jin Y, Knudsen E, Wang L, Bryceson Y, Damaj B, Gessani S, Maghazachi AA (2003). Sphingosine 1-phosphate is a novel inhibitor of T-cell proliferation. Blood.

[B29] Miller JS, Oelkers S, Verfaillie C, McGlave P (1992). Role of monocytes in the expansion of human activated natural killer cells. Blood.

[B30] Dunne J, Lynch S, O'Farrelly C, Todryk S, Hegarty JE, Feighery C, Doherty DG (2001). Selective expansion and partial activation of human NK cells and NK receptor-positive T cells by IL-2 and IL-15. J Immunol.

[B31] Walker MR, Kasprowicz DJ, Gersuk VH, Benard A, Van Landeghen M, Buckner JH, Ziegler SF (2003). Induction of FoxP3 and acquisition of T regulatory activity by stimulated human CD4+CD25- T cells. J Clin Invest.

[B32] Klenerman P, Cerundolo V, Dunbar PR (2002). Tracking T cells with tetramers: new tales from new tools. Nat Rev Immunol.

[B33] Poupot M, Fournie JJ (2004). Non-peptide antigens activating human Vgamma9/Vdelta2 T lymphocytes. Immunol Lett.

[B34] Mestel DS, Assaf C, Steinhoff M, Beyer M, Moebs M, Sterry W (2008). Emerging drugs in cutaneous T cell lymphoma. Expert Opin Emerg Drugs.

