# Supplementary material for: Regulatory activity of azabisphosphonate-capped dendrimers on human CD4+ T cell proliferation enhances ex-vivo expansion of NK cells from PBMCs for immunotherapy
Source: J Transl Med. 2009 Sep 24;7:82. doi: 10.1186/1479-5876-7-82 (PMC2761872; doi:10.1186/1479-5876-7-82)
Supplement: Additional file 1 — 3a-G1 does not affect NK cell cytotoxicity. Standard 4 h 51Cr-release assay determining the specific lysis of K562 pulsed cells by PBMCs cultured for two weeks in the presence or in the absence of 3a-G1. Effector/Target ratio was normalized according to the percentage of NK cell present in each culture. [file 1479-5876-7-82-S1.pdf]

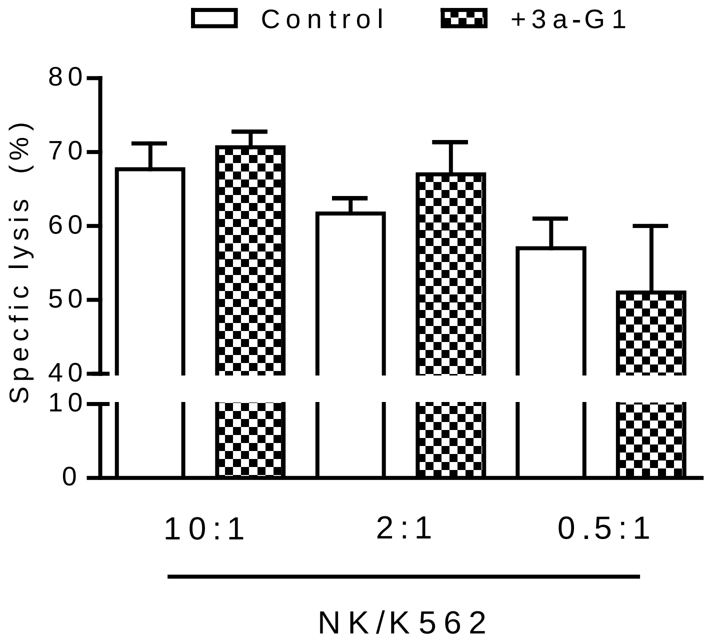

Additional file 1: 3a-G1 does not affect NK cell cytotoxicity. Standard 4h ^{51}Cr-release assay was performed to determine the specific lysis of K562 pulsed cells by PBMCs cultured for two weeks in the presence or in the absence of 3a-G1. Effector/Target ratio was normalised according to the percentage of NK cell present in each culture.
